# Diabetes: Non-Invasive Blood Glucose Monitoring Using Federated Learning with Biosensor Signals

**DOI:** 10.3390/bios15040255

**Published:** 2025-04-16

**Authors:** Narmatha Chellamani, Saleh Ali Albelwi, Manimurugan Shanmuganathan, Palanisamy Amirthalingam, Anand Paul

**Affiliations:** 1 Faculty of Computers and Information Technology, University of Tabuk, Tabuk 71491, Saudi Arabia; narmatha@ut.edu.sa (N.C.);; 2 Department of Pharmacy Practice, Faculty of Pharmacy, University of Tabuk, Tabuk 71491, Saudi Arabia; 3 Biostatistics and Data Science Department, The School of Public Health, LSU Health Sciences Center New Orleans, New Orleans, LA 70112, USA

**Keywords:** diabetes management, non-invasive blood glucose monitoring, photoplethysmography (PPG), deep neural networks (DNNs), machine learning, federated learning (FL), healthcare, Clarke error grid analysis

## Abstract

Diabetes is a growing global health concern, affecting millions and leading to severe complications if not properly managed. The primary challenge in diabetes management is maintaining blood glucose levels (BGLs) within a safe range to prevent complications such as renal failure, cardiovascular disease, and neuropathy. Traditional methods, such as finger-prick testing, often result in low patient adherence due to discomfort, invasiveness, and inconvenience. Consequently, there is an increasing need for non-invasive techniques that provide accurate BGL measurements. Photoplethysmography (PPG), a photosensitive method that detects blood volume variations, has shown promise for non-invasive glucose monitoring. Deep neural networks (DNNs) applied to PPG signals can predict BGLs with high accuracy. However, training DNN models requires large and diverse datasets, which are typically distributed across multiple healthcare institutions. Privacy concerns and regulatory restrictions further limit data sharing, making conventional centralized machine learning (ML) approaches less effective. To address these challenges, this study proposes a federated learning (FL)-based solution that enables multiple healthcare organizations to collaboratively train a global model without sharing raw patient data, thereby enhancing model performance while ensuring data privacy and security. In the data preprocessing stage, continuous wavelet transform (CWT) is applied to smooth PPG signals and remove baseline drift. Adaptive cycle-based segmentation (ACBS) is then used for signal segmentation, followed by particle swarm optimization (PSO) for feature selection, optimizing classification accuracy. The proposed system was evaluated on diverse datasets, including VitalDB and MUST, under various conditions with data collected during surgery and anesthesia. The model achieved a root mean square error (RMSE) of 19.1 mg/dL, demonstrating superior predictive accuracy. Clarke error grid analysis (CEGA) confirmed the model’s clinical reliability, with 99.31% of predictions falling within clinically acceptable limits. The FL-based approach outperformed conventional deep learning models, making it a promising method for non-invasive, privacy-preserving glucose monitoring.

## 1. Introduction

Diabetes is a long-term metabolic [1,2,3] condition that sufferings millions of individuals globally. The issue cascades under hyperglycemia or increased blood glucose, which is carried on through either the physique’s struggle with insulin or inadequate production of insulin [4]. Diabetes might lead to severe complications like kidney damage, heart disease, nerve issues, and vision loss if it is not managed [5,6,7]. According to the WHO (World Health Organization), the occurrence of diabetes has amplified significantly, with about 422 million people globally living with the disease in 2021 [8]. This quantity is anticipated to rise to over 700 million through 2045 [4], typically due to inactive lifestyles, urbanization, aging populations, and unhealthy eating patterns [9]. For diabetes to be efficiently accomplished, the blood sugar level must be frequently monitored. Conservative approaches, such as finger-prick examination, entail painful and difficult skin punctures to gather little blood samples [10]. Owing to this pain, patients typically do not follow suggested monitoring schedules [11]. Researchers [12,13,14] are utilizing non-invasive proxies that provide more accessibility and real-time tracking capabilities to address these concerns. One such fortunate method is PPG, a device that tests variations in blood volume by analyzing how light is engrossed or replicated by blood vessels [15]. Because GLs alter physiological features such as blood flow and tissue transparency, PPG signals can capture these changes [16,17]. It can be assessed without taking a blood trial straight using sophisticated calculations [18]. By enhancing patient adherence and enabling continuous monitoring, this non-invasive technique improves glucose control and reduces the risk of diabetes-related complications.

**Figure 1:**
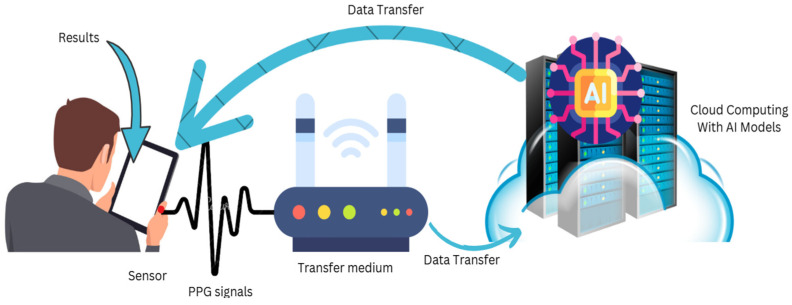
General architecture of non-invasive blood glucose monitoring using PPG.

PPG is a versatile method that extends beyond traditional BGM and can be applied in various healthcare settings. Researchers have scrutinized the use of physiological signals, similar abnormal blood flow patterns, and amplified heart rates for stress recognition. Likewise, PPG-based features, with waveform pulse and shape transit time, were utilized to assess blood pressure, enabling the measurement of systolic and diastolic pressure without the need for predictable cuff-based systems. These applications highlight PPG’s potential as a practical tool for evaluating digital health (Figure 1).

The consistency and accuracy of PPG-based BGM were significantly enhanced through the integration of artificial intelligence (AI) and machine learning (ML) [19,20,21,22,23,24,25]. These methods facilitate the identification of complex patterns in signals from PPG to correlate with fluctuations in blood sugar levels, resulting in the development of predictive models that accommodate the physiological differences of each individual. Additionally, AI-driven methodologies enhance the modification of BGM systems, thus improving their efficiency and longevity. To improve the precision of BGL models for forecasts, certain studies have concentrated on feature extraction methodologies [24,25]. To capture physiological differences associated with glucose levels, these methods analyze PPG data and extract critical data, for instance, waveform features, pulse rate, and pulse amplitude [12,14]. Experts are required to increase reliability and precision by adding these features to replicas for forecasting. Other variables like HbA1c (i.e., glycated hemoglobin) levels were also combined into the models of several types of research. It can be an obliging component in the BGL forecast because it is the mean BGL for the preceding two to three months. Nonetheless, there are risks to utilizing HbA1c alone, as it fails to recognize rapid or short-term alterations in blood sugar levels, an issue that real-time PPG monitoring resolves. Numerous investigations were examined using raw PPG signals alone for BGL evaluation [26]. Instead of manually creating novel features, they used the PPG waveform’s previous information to obtain descriptive observations directly. The above method streamlines the process and lowers computer complexity without sacrificing glucose level estimation precision.

A previous study has investigated the use of PPG for blood glucose forecasting; many of these investigations employed DNNs to provide highly accurate estimates. However, DNN models require enormous and diverse datasets, which are frequently distributed across multiple hospitals. The transfer of information is further restricted by rigorous data privacy laws and security issues, which reduces the efficacy of centralized ML techniques. Federated Learning (FL) tackles this problem in this work by helping several healthcare organizations work together to train a global model while maintaining the privacy and security of patient data. To wrap up the introduction, the main findings of this study are emphasized, distinguishing it from prior lessons and accentuating its consequence in the domain of non-invasive BGM.

- To smooth the PPG signal and remove base drift while data preprocessing, the CWT method is utilized;
- To select features, the PSO method is utilized to enhance classification accuracy. There are some factors used to train the FL classifier;
- The methodology has been evaluated on two datasets, one from normal physiological conditions and the other from anesthesia, proving the model’s adaptability in multiple situations. The results of this research indicated 99.31% clinical acceptability with a mean RMSE of 19.1 mg/dL, 76.8% precision in the CEGA’s A zone, and 22.7% accuracy in its B zone. Furthermore, with a computation time of just six seconds, the top-performing model is effectively implemented on an embedded device;
- These findings demonstrate the potential for real-world application in compact and connected health monitoring devices and verify the effectiveness of sophisticated PPG segmentation strategies.

This document examines the prediction of BGL utilizing PPG data. Section 2 reviews the current literature in this domain, highlighting various modeling methodologies. Section 3 outlines the datasets used, the preprocessing, segmentation, and feature selection strategies employed, as well as the implementation and evaluation of the federated learning (FL) framework for both PPG and BGL data. Section 4 delineates the performance measures and contrasts several methods of classification. The Conclusion encapsulates the principal findings and proposes directions for upcoming investigation.

## 2. Related Work

Numerous research has examined the forecast of BGL from PPG signals, utilizing various approaches. Zeynali et al. [27] observed a non-invasive approach for BGM via PPG with a novel 1-s signal segment algorithm. This research assesses the accuracy, reliability, and adaptability of three advanced deep learning models across various datasets. The suggested 1-s segmentation technique improves precision and processing velocity relative to conventional 10-s segments. The optimized model, implemented on an embedded device, provides hasty blood glucose estimations in about 6.4 s. Evaluations were conducted on datasets about surgery, anesthesia, and normal physiological settings, attaining a 19.7 mg/dL RMSE with complete clinical acceptance. These findings illustrate the capability of this approach for accurate, efficient, and non-invasive diabetes control.

Chen, S. et al. [28] introduced an advanced multi-view cross-fusion transformer (MvCFT) network based non-invasive technique for measuring BGLs utilizing PPG signals aimed to refining diabetes monitoring and supervision. It combines signal preprocessing optimization by MvCFT a for precise glucose assessment. The MSWF (multi-size weighted fitting) filtering scheme upholds vital signal features, while spatial position-based moving features enhance physiological pattern finding. A CVFF (cross-view feature fusion) component joins in concepts from numerous views to improve precision. The model experienced evaluation with 260 clinical members, resulting in a f 0.659 mmol/L MAE, 1.129 mmol/L RMSE, and a precision of 87.89% in Zone A of the CEG, thus affirming its consistency without any clinical danger.

Rostami et al. [29] suggested a solution for real-time stress recognition with a DL model known as LSTM (long short-term memory). The model observes PPG signals from the WESAD dataset to control an individual’s stress level. It analyses the data by segmenting it into smaller temporal intervals and subsequently uses ML to precisely classify stress levels. The researchers employed advanced optimization methods from TensorFlow, pruning, including PTQ (post-training quantization) and QAT (quantization aware training), to expand the system’s competence and decrease its size while maintaining excellent precision. The finished model is very lightweight, maintaining an accuracy of 87.76%.

Satter et al. [30] presented a non-invasive technique for determining BGL via PPG signals from wrist-mounted devices. It observes PPG waveform features, encompassing the AC/DC ratio and features based on intrinsic mode functions (IMF) attained using empirical mode decomposition (EMD). Feature importance-based selection was utilized to expand model accuracy. CatBoost, XGBoost, LightGBM, and random forest-based ML models were used, and CatBoost consistently exhibited superior performance. The optimal model attained a 0.96 Pearson’s correlation value, a 0.08 MSE, a 0.92 R^2^ score, and 8.01 MAE (mean absolute error) while employing the 50 most significant features. All replicas established statistically significant associations (*p*-values < 0.001).

Chowdhury et al. [31] introduced MMG-net, a multi-modal system designed for the estimation of BGLs via a non-invasive monitoring model that integrates data from wearable sensors, such as EDA (electrodermal activity), PPG, dietary logs, and skin temperature. MMG-Net achieves remarkable precision, with a 13.51 mg/dL MAE; 99.43% of forecasts are under clinically acceptable ranges. It surpasses current systems, providing a promising and non-invasive alternative for hyperglycemia supervision.

Nie et al. [19] presented a non-invasive expertise for enumerating BGLs with a near-infrared camera, thus addressing the restrictions of invasive approaches that can be measured by analyzing reflected light. In this approach, 26 pulse wave characteristics are examined, and six features with strong correlations to BGLs are selected. Four ML schemes like PLS (partial least squares), PCR (principal component regression), RFR (random forest regression), and SVR (support vector regression), were evaluated, with RFR demonstrating higher performance and nominated for the final extrapolative system. Experimental findings, evaluated via CEGA, establish good precision and robust harmony with conservative glucose monitors. This non-invasive system offers a promising and painless alternative for focusing BGLs.

Susana et al. [32] examined a non-invasive method based on a support vector machine (SVM) for BGM via PPG. Time-frequency methods are utilized to analyze the data from 219 people at Guilin People’s Hospital, recognizing variables like temporal frequency and spectrum entropy. Three versions of PPG data were assessed using ML systems, achieving the maximum precision of 91.3% in 9 s. The findings highlight the promise of PPG-based management as a non-invasive complement to invasive techniques; however, further improvements are essential. Vargová et al. [33] investigated an SVM and random forest (RF) based non-invasive BGM utilizing PPG signals from smart gadgets. A smart bracelet and smartphone were used to collect data from 16 participants, with invasive GLs helping as benchmarks. After preprocessing and feature selection, ML models were implemented. SVM and RF demonstrated the maximum accuracy in glycemic classification, attaining 75–76% accuracy. RF had superior performance in forecasting GLs, achieving MAEs of 1.37 mmol/L (smartphone) and 1.25 mmol/L (wristband). Susana et al. [34] proposed a non-invasive, cost-effective technique for BGM utilizing PPG signals and ML. PPG data were attained from a finger sensor and analyzed using AI for glucose categorization. ML was favored over deep learning (DL) to improve competence. A dataset of 400 samples was classified as normal or diabetic according to clinical norms. The ensemble bagged trees approach exhibited the highest reliability of 98% among the evaluated replicas, underscoring its efficiency in diabetes recognition.

Yen et al. [35] introduced a BPNN (back-propagation neural network) based non-invasive BGM system that employs dual-wavelength PPG and bioelectrical resistivity monitoring to progress exactness while removing the pain associated with invasive methods. PPG signals were altered into statistical features, though bioelectrical impedance data, encompassing real and imaginary mechanisms, time, and amplitude across 11 frequencies, were analyzed using principal component analysis (PCA). These, in aggregation with seven physiological features, served as inputs for a BPNN to estimate glucose levels. Information from 40 participants corroborated the system, resulting in an MSE of 40.736, an RMSE of 6.3824, and an R^2^ of 0.997.

Based on the preceding studies (Table 1), establishing reliable models for BGL estimates utilizing PPG signals is difficult, mainly as data is scarce. Whereas the BGL estimate shows potential via PPG, the model’s stability and precision depend on the size and diversity of the training information. The absence of data in small-scale studies restricts adaptability, whereas big datasets, regardless of their advantages, complicate data handling and computing and can generate biases. Efficient algorithms and meticulous management of confounding variables are crucial for creating scalable and dependable non-invasive BGL estimate models. To tackle this issue, the proposed solution in this research is the FL.

## 3. Proposed Methodology

**Figure 2:**
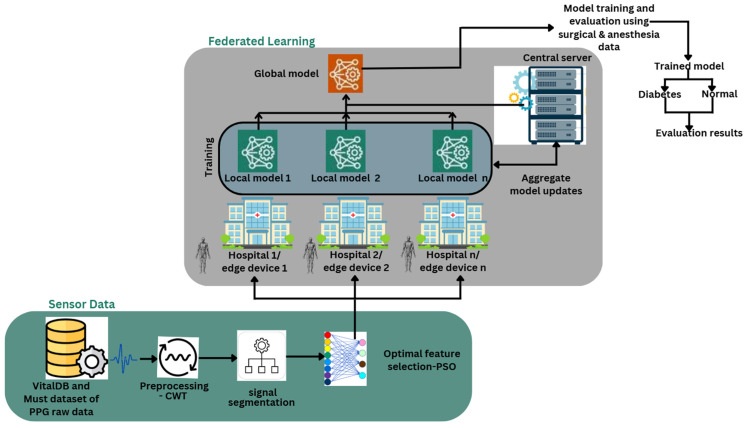
Proposed architecture of non-invasive BGM using PPG Signals by federated learning.

Figure 2 shows the entire procedure of the suggested FL framework for non-invasive BGL monitoring. Raw PPG signals are first obtained from VitalDB and MUST, two publically accessible datasets. Unprocessed PPG recordings from 82 people, 45 from the VitalDB dataset, and 37 from the MUST dataset, representing a range of physiological and demographic characteristics, are included in these datasets. However, noise from motion artifacts, inaccurate sensors, and ambient disturbances frequently affect raw PPG signals. For precise extraction and sorting of features, preprocessing methods like CWT are used to improve signal quality and denoise the signal. Further to preprocessing, the purified signal is partitioned into smaller temporal segments to extract significant patterns. Because PPG signals comprise time-series data, segmentation is essential for identifying significant patterns for further examination. Feature selection is performed using PSO to enhance system reliability and identify the most relevant features, thus lowering computational complexity and increasing precision in classification. The PPG data is distributed among numerous hospitals, typically utilizing edge devices for local model training instead of sending raw patient data to a centralized database. This federated learning approach prioritizes security and privacy of data, preventing unauthorized access to sensitive patient data. Each hospital formulates a localized model employing PSO-optimized PPG characteristics, allowing the model to identify patterns related to diabetes. Hospitals communicate only updated models to a centralized server for aggregation instead of supplying raw data. The central server aggregates inputs from several healthcare organizations and integrates them into a unified model, exploiting the diverse datasets from distinct hospitals. Employing federated learning improves the model’s generalization and reliability, later augmenting its accuracy in diabetes diagnosis. The global model undergoes additional training and validation using data from surgical and anesthetic patients to improve its accuracy and ensure reliable performance in real clinical settings. After completion of training, the model can expertly classify patients, such as normal or diabetic, with risk levels varying from minimal to extreme risk.

### 3.1. Dataset Description

VitalDB: The VitalDB databases [27] serves as a high-resolution repository of biosignals designed for the examination of patient treatment during surgical and anesthetic interventions. This includes critical vital signs such as oxygen saturation, blood pressure, heart rate, and body temperature. An important characteristic is the integration of PPG signals, which are cardiovascular data and enable real-time BGL monitoring through the TramRac4A device. The dataset consists of 6388 participants, allocated 70% for training, 15% for verification, and 15% for examination, rendering it a significant asset for biosignal assessment and clinical studies.

MUST: The MUST dataset [27] was compiled by the Digital Systems Research Team at the University of Science and Technology. The dataset consists of 67 raw PPG signals, each accompanied by supplementary information such as age, gender, and invasively measured BGLs. This dataset is especially significant for research in non-invasive BGM for health diagnosis. It is utilized exclusively for testing reasons, not for model training.

### 3.2. Preprocessing and Signal Segmentation

PPG is prevalent; nonetheless, unprocessed PPG signals frequently exhibit motion distortions, sensor noise, and baseline drift, which may diminish the precision of BGL assessment. The continuous wavelet transform (CWT) is a proficient preprocessing method for denoising, baseline elimination, and feature extraction, facilitating enhanced BGM. CWT converts a time–domain into a time–frequency depiction, facilitating the analysis of PPG. The CWT of a signal ip(t) is expressed as Equation (1):

(1) $$WC_{ip}\left(x,y\right)=\int_{-\infty }^{\infty }ip\left(t\right)\psi^{*}\left(\frac{t-y}{x}\right)dt$$

where ip(t) is the input PPG signal, $WC_{ip}\left(x,y\right)$ represents the wavelet coefficient at scale x and time shift y, x controls the frequency resolution, and y defines the location of the wavelet in time, $\psi^{*}\left(\frac{t-y}{x}\right)$ is represents the compound conjugate of the mother wavelet process and is defined as Equation (2):

(2) $$\psi_{x,y}\left(t\right)=\frac{1}{\sqrt{\left|x\right|}}\psi \left(\frac{t-x}{y}\right)$$

where $\frac{1}{\sqrt{\left|x\right|}}$ ensures energy normalization, $\left(\frac{t-x}{y}\right)$ represents the scales and translates the wavelet in time. In this context, the Morlet and Mexican hat wavelets are active as mother wavelets. The Morlet wavelet is employed for the detection of fluctuations and peaks in PPG data and is defined as Equation (3):

(3) $$\psi \left(t\right)=e^{-t^{2}}e^{j2\pi f_{0}t}$$

where $f_{0}$ is ensured the central frequency.

The Mexican hat is employed to identify abrupt transitions and alterations in PPG, which is the second derivative of a Gaussian function defined as Equation (4).

(4) $$\psi \left(t\right)=\left(1-t^{2}\right)e^{t^{2}/2}$$

This wavelet is effective for identifying pulse waveforms affected by glucose variations.

(A) CWT-based Noise reduction

Noise and motion distortions in PPG signals can be eliminated through thresholding of wavelet coefficients. The denoising procedure consists of the following steps:

Step 1: PPG signals decomposed using CWT to compute wavelet coefficient $WC_{ip}(x,y)$ at different scales and shifts.
Step 2: Soft thresholding is applied to remove noise, as shown in Equation (5)
  (5) $$WC_{ip}^{\prime }\left(x,y\right)=\left\{\begin{array}{c} sign\;\left(WC_{ip}\left(x,y\right)\right)\left(\left|WC_{ip}\left(x,y\right)\right|-T\right),\;if\;\left|WC_{ip}\left(x,y\right)\right|>T\\ \;\;0,\;\;otherwise \end{array}\right.$$
  where $sign\left(.\right)$ is the sign function, which conserves the coefficient’s sign after thresholding. T (threshold) represents which wavelet coefficients should be retained or suppressed, and is calculated by using Donoho’s universal threshold and is defined as Equation (6):
  (6) $$T=\sigma \sqrt{2logN}$$
  where the noise standard deviation is σ, N represents the number of data points.
Step 3: Inverse CWT(ICWT) is used to reconstruct the denoised (d) signal, as shown in Equation (7)
  (7) $$ip_{d}\left(t\right)=\int_{x_{min}}^{x_{max}}\int_{y_{min}\;}^{y_{max}}WC_{ip}^{\prime }\left(x,y\right)\psi_{x,y}\left(t\right)dxdy$$

(B) Segmentation using Adaptive Cycle-Based Segmentation (ACBS)

**Figure 3:**
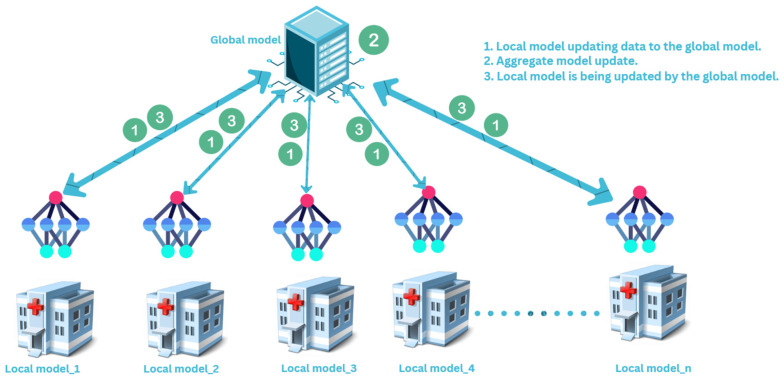
Proposed FL model architecture.

ACBS is an advanced method employed to segment PPG signals into distinct heartbeat cycles, facilitating a more comprehensive analysis of cardiovascular factors, especially concerning physiological alterations such as fluctuations in glucose levels, and the segmented PPG signal is shown in Figure 3. The process begins with the preprocessing of the PPG signal, which includes methods such as CWT to reduce noise and enhance signal quality. The segmentation approach subsequently identifies R-peaks, indicating the peak of each cardiac cycle. Upon identifying these peaks, the approach defines the boundaries of each cycle, typically positioning them at the midpoint among consecutive R-peaks to capture the whole waveform of a heartbeat. In both the first and final cycles, if a preceding or succeeding R-peak is absent, preset time intervals are utilized alternatively. The PPG signal is subsequently divided according to these limits, and to maintain consistency between cycles, normalizing techniques such as resampling to a uniform length and amplitude scaling are utilized. This normalization approach reduces fluctuations in heart rate and signal quality, ensuring consistency in the resultant cycles for improved feature extraction and analysis. By focusing on individual cardiac cycles, ACBS provides a comprehensive analysis of PPG signals, aiding the detection of subtle morphological changes that may indicate physiological variations, which are associated with glucose levels and other health concerns. The following sequential process is detailed below:

Step 1: The unprocessed PPG signal is subjected to preprocessing via the execution of CWT. The denoised PPG signal is displayed following filtration in Equation (8).

(8) $$ip^{\prime }\left(t\right)=ip\left(t\right)-n\left(t\right)$$

where n(t) represents the noise components removed during preprocessing.

Step 2: Detection is performed using a thresholding method. A simple approach involves identifying local maxima in the first derivative, where $ip^{\prime }\left(t\right),$ as defined in Equation (9).

(9) $${Rp}_{k}=\arg\;\begin{array}{c} max\\ t_{k} \end{array}\left(\frac{dip^{\prime }}{dt}\right)\;\;\;\forall t_{k}\in \left[t_{k-1},t_{k+1}\right]$$

where ${Rp}_{k}$ represents the location of the kth R-peak.

Step 3: Following R-peak identification, the limits of each cardiac cycle are established. The start $\left(St_{k}\right)$ and $\left(E_{k}\right)$ end of each cycle are set midway between consecutive R-peaks, as shown in Equation (10)

(10) $$St_{k}=\frac{{Rp}_{k-1}+{Rp}_{k}}{2},\;E_{k}=\frac{{Rp}_{k}+{Rp}_{k+1}}{2}$$

For the first and last cycles, where there is no preceding or following R-peak, fixed time windows ($T_{f})$ are used and defined as Equation (11)

(11) $${St}_{1}={Rp}_{1}-T_{f},\;E_{N}={Rp}_{N}+T_{f}$$

where the total amount of perceived R-peaks is defined as *N*.

Step 4: Each heartbeat cycle $Cy_{k}(t)$ is extracted from the PPG signal using Equation (12)

(12) $$Cy_{k}\left(t\right)=ip^{\prime }\left(t\right),\;\forall t_{k}\in [{St}_{k},E_{k}\;]$$

Step 5: Heartbeats exhibit variability in duration and amplitude; thus, normalization is utilized to achieve consistent representation. Each cycle ${Cy}_{k}(t)$ is resampled to standard length Lg using interpolation and is defined in Equation (13)

(13) $$Cy_{k}^{norm}\left(n\right)=Cy_{k}\left(St_{k}+\frac{n}{Lg}\left(E_{k}-St_{k}\right)\right),\;n\in \left[1,Lg\right]$$

To ensure consistency, the PPG signal amplitude is scaled between 0 and 1 and is shown in Equation (14).

(14) $$Cy_{k}^{norm}\left(n\right)=\frac{Cy_{k}\left(n\right)-\min\;(Cy_{k})}{\max\;\left(Cy_{k}\right)-\min\;(Cy_{k})}$$

### 3.3. Feature Selection Using PSO

Following the segmentation and normalization of individual cardiac cycles using ACBS, the next crucial step involves selecting the most relevant features [36,37] that represent physiological alterations, particularly those associated with glucose fluctuations. Given that every heartbeat cycle encompasses a substantial amount of information, it is essential to determine the most pertinent features, such as time-domain features (e.g., pulse amplitude, area under the curve, pulse width, inter-pulse interval, and slope), frequency-domain features (e.g., dominant frequency, power spectral density, and heart rate variability), and nonlinear features (e.g., entropy, fractal dimension, and Poincaré plot), as this enhances both efficiency and accuracy. To do this, PSO is utilized, a technique derived from the collective actions of avian and aquatic species. In this approach, each “particle” signifies a potential combination of chosen attributes from the cardiac cycles. The worth of each set is assessed via training a model, like a classifier, and evaluating its performance (e.g., accuracy or error rate). The particles subsequently traverse various feature combos, modifying their trajectory according to their optimal outcomes (i.e., local best) and the superior overall outcome within the group (i.e., global best). This method allows the swarm to systematically find the optimal feature set, improving the precision of models while reducing irrelevant information. By selecting an optimal and compact set of characteristics, PSO enhances model efficacy, reduces computational needs, and increases the ability to predict glucose variations or classify health conditions.

The system used 30 particles for PSO-based feature selection in this investigation. To balance exploration and exploitation, the inertia weight (wt) was chosen at 0.729. Equation (21) indicates that the cognitive (co_1_) and social (co_2_) coefficients were set at 1.49 to guide particles using personal (pBest) and global (gBest) experiences. After 50 iterations, the method converged by minimizing RMSE using Equation (20)’s fitness function. These standard parameter choices help ensure efficient and effective feature subset optimization.

The approach used for feature selection utilizing PSO is delineated below:

Step 1: Extracted features representation: Every segmented and regularized cardiac cycle $Cy_{k}^{norm}\left(n\right)$ comprises multiple features collected for examination. Let $Fe=\left\{fe_{1},fe_{2},\ldots fe_{d}\right\}$ define the set of extracted features, where d is represents the total number of features per heartbeat cycle. Potential features may encompass:

Feature Vector: After extracting these *d* features, the *i*-th heartbeat cycle is signified via a feature vector is defined in Equation (15):

(15) $$A_{i}=[fe_{i1},\;fe_{i2},\;fe_{i3}\;\ldots ,\;fe_{id}{]}^{T}$$

where *f_ji_* signifies the *j*-th characteristic extracted from the *i*-th heartbeat, d signifies the total quantity of features extracted for each heartbeat cycle. For the N-segmented heartbeat cycles, the feature matrix is defined as Equation (16):

(16) $$A=\left[\begin{array}{ccc} fe_{1,1} & fe_{1,2} & \begin{array}{cc} \ldots & fe_{1,d} \end{array}\\ fe_{2,1} & fe_{2,2} & \begin{array}{cc} \ldots & fe_{2,d} \end{array}\\ \begin{array}{c} \vdots \\ fe_{N,1} \end{array} & \begin{array}{c} \vdots \\ fe_{N,2} \end{array} & \begin{array}{cc} \begin{array}{c} \ddots \\ \ldots \end{array} & \begin{array}{c} \vdots \\ fe_{N,d} \end{array} \end{array} \end{array}\right]$$

where $f_{k,j}$ signifies the j-th characteristic of the k-th heartbeat cycle.

Step 2: Representation of Particles: Each particle in the PSO represents a candidate feature subset from the feature space in Equation (17):

(17) $$Sb_{i}=\{sb_{1},sb_{2},\ldots ,sb_{d}\},sb_{j}\in \{0,1\}$$

if $sb_{j}=1$ means the feature is selected, and 0 means discarded.

Step 3: Initialization of Particles: Initialize X particles randomly, where each particle’s position (Pp) is represented by Equation (18):

(18) $$Pp_{i}=\left(sb_{1}^{i},\;sb_{2}^{i},\ldots ,sb_{d}^{i}\right),\;\forall i\in \{1,2,\ldots ,X\}$$

Each particle has a velocity vector initialized in Equation (19):

(19) $$Ve_{i}=(ve_{1}^{i},ve_{2}^{i},\ldots ,ve_{d}^{i},\;)$$

where $Ve_{j}^{i}$ determines the probability of selecting or discarding a feature.

Step 4: Evaluation of fitness function: The fitness (*Ft*) function evaluates the quality of every particle’s feature collection. A prevalent method involves training a prediction model with the chosen features and assessing its efficacy. The fitness function can be articulated as Equation (20):

(20) $$Ft\left(Sb_{i}\right)=\alpha \times acc\left(Sb_{i}\right)-\beta \times \frac{\left|Sb_{i}\right|}{d}$$

where acc($Sb_{i}$) is the classification accuracy of the model trained with the feature subset $Sb_{i}$ and $\left|Sb_{i}\right|$ is the number of selected features in $Sb_{i}$, α and β are weighting factor that balances model performance, and $Sb_{i}$ feature size.

Step 5: Each particle updates its velocity and position using the following Equation (21).

(21) $$Ve_{j}^{i}\left(t+1\right)=wt.Ve_{j}^{i}\left(t\right)+co_{1}.ra_{1}.\left(pBest_{j}^{i}-Sb_{j}^{i}\right)+co_{2}.ra_{2}.(gBest_{j}-Sb_{j}^{i})$$

where wt is referred to the inertia weight that controls the exploration vs. exploitation, $co_{1},co_{2}$ defines the cognitive and social coefficients, $ra_{1},ra_{2}$ is the Random values in [0, 1], $pBest_{j}^{i}$ refers to the Best place of the i-th atom, $gBest_{j}$ is the global best location found via the swarm.

Step 6: Updating the position.

Using a sigmoid function to map velocity to a probability as predicted in Equation (22):

(22) $$Sb_{j}^{i}\left(t+1\right)=\left\{\begin{array}{c} 1,\;\;if\;\sigma \left(Ve_{j}^{i}\left(t+1\right)\right)>ra\\ 0,\;\;otherwise\; \end{array}\right.$$

where $\sigma \left(Ve_{j}^{i}\right)=\frac{1}{1+e^{-Ve_{j}^{i}}}$.

Step 7: Update personal and global best is defined as in Equations (23) and (24):

(23) $$pBest_{i}=Sb_{i},\;\;if\;Ft\left(Sb_{i}\right)>Ft(pBest_{i})$$

(24) $$gBest=\arg\;\begin{array}{c} max\\ i \end{array}Ft\left(pBest_{i}\right)$$

Step 8: Check the stopping criterion: if there is no improvement for A consecutive iterations, terminate the process.

### 3.4. Classification Using Federated Learning

Following feature selection via PSO, federated learning (FL) facilitates collective and confidential monitoring of BGL via PPG signals. Every medical facility or wearable gadget develops a localized model utilizing the chosen PPG features to determine the correlation between PPG signals and blood glucose levels. In lieu of transmitting confidential patient information, only updated models (i.e., improved parameters) are sent to a central server when the results are pooled through federated averaging (FA) to formulate a global model. It leverages varied datasets from various places, enhancing its generality and precision (Figure 3).

The updated model is then transmitted to designated devices for further local training, perpetually enhancing its performance across numerous iterations. Federated learning enhances the precision, privacy, and scalability of real-time BGM by preserving personal data and deriving insights from a heterogeneous population, thus creating a reliable solution for diabetes care. The model performs both regression and classification by the prediction of continuous BGLs and their categorization into hypoglycemia, normoglycemia, or hyperglycemia. FL enables training across several devices while safeguarding raw data confidentially, later ensuring privacy and security. The model utilizes a multi-task learning (MTL) framework to optimize both tasks, hence improving overall accuracy. Statistical optimization techniques, including FL and appropriate loss functions, enhance effective model training, aligning continuous predictions with categorical classifications for better diabetes management.

Multi-Task Learning (MTL) in FL: Two loss functions are used to train the model.

Regression Loss $L_{r}$ with MSE and is expressed as Equation (26)

(25) $$L_{c}=\frac{1}{N}\sum_{i=1}^{N}\sum_{cl=1}^{CL}{\left(P_{i,cl}\log\;{\hat{P}}_{i,cl}\right)}^{2}$$

(26) $$L_{r}=\frac{1}{N}\sum_{i=1}^{N}{\left(P_{i}-\hat{P_{i}}\right)}^{2}$$

where $P_{i}$ defines the true BGL value, and $\hat{P_{i}}$ refers to the predicted BGL value.

Classification Loss ($L_{c}$) with categorical cross-entropy (CCE) is defined as Equation (25). Where $P_{i,cl}$ is defined as a one-hot encoded ground truth for class (cl) and ${\hat{P}}_{i,cl}$ is the predicted probability for class (cl).

Combined Loss for Joint Learning: The total loss function ($L_{t}$) is a weighted sum is given in Equation (27):

(27) $$L_{t}=\frac{1}{N}\lambda_{1}L_{r}+\lambda_{2}L_{cl}$$

where $\lambda_{1}$ and $\lambda_{2}$ are controls the importance of each task.

Each hospital or wearable device $Hp_{i}$ trains a local model using its dataset $Ds_{i}=(A_{i},B_{i})$. The aim is to minimize a local loss function $Lt_{i}\left(W\right),$ as defined in Equation (28):

(28) $$Lt_{i}\left(Mp\right)=\frac{1}{N_{i}}\sum_{j=1}^{N_{i}}l\left(f\left(a_{j};Mp\right),b_{j}\right)$$

where Mp represents the model parameters, $l\left(f\left(a_{j};Mp\right),b_{j}\right)$ is the loss function, and $f\left(a_{j};Mp\right)$ is the model output, predicting blood glucose levels. The local update is performed using gradient descent is defined as Equation (29).

(29) $${Mp}_{i}^{t+1}={Mp}_{i}^{t}-\eta \nabla Lt_{i}({Mp}_{i}^{t})$$

where η is the learning rate.

Subsequently, the model is transmitted to the server for updating. Rather than transmitting raw data, every hospital or device conveys solely the model modifications to the central server for aggregation and is referred to in Equation (30):

(30) $$∆{Mp}_{i}={Mp}_{i}^{t+1}-{Mp}_{i}^{t}$$

Aggregation is employed to construct a global model by FA. The central server consolidates model updates from K participating clients (e.g., hospitals or devices) to formulate a global model defined as Equation (31):

(31) $$Mp^{t+1}=\sum_{i=1}^{K}\frac{N_{i}}{N}Mp_{i}^{t+1}$$

where N represents the aggregate amount of data from all clients. The aggregation assigns weights to each hospital’s output according to the size of the dataset. The updated global model is thereafter disseminated to each client for further local training. The procedure is reiterated for several training iterations until the model comes together. The global function that is optimized in FL is expressed mathematically as Equation (32).

(32) $$\begin{array}{c} min\\ Mp \end{array}\sum_{i=1}^{K}\frac{N_{i}}{N}Lt_{i}\left(Mp\right)$$

After training, the final global model is used for classification. Given a new PPG feature vector a, the BGL prediction is Equation (33):

(33) $$\hat{b}=\arg\;\begin{array}{c} max\\ b\in \left\{0,1\right\} \end{array}f\left(a;Mp^{f}\right)$$

where $Mp^{f}$ is the final optimized model.

The balance between clinical accuracy and useful deployment in resource-constrained instances appears in the model’s RMSE and MAE performance. The STM32-based embedded solution allows for real-world use for continuous, accessible BGM in a variety of scenarios, in contrast to complicated offline systems with few patients. Using a specialized sensor, the procedure starts with the capture and preprocessing of PPG signals. For privacy, FL is used to train and validate deep learning models on a remote server equipped with a 3090 GPU. Feature selection based on PSO improves efficiency and accuracy. To be deployed on the STM32H743IIT6 MCU, the optimized model is quantized, punched, and compiled into a binary format [27]. It is moved to external flash memory (W25Q256) and uses the “execute in place” (XIP) feature to run in real time, guaranteeing constant, non-intrusive monitoring.

## 4. Results and Discussion

**Figure 4:**
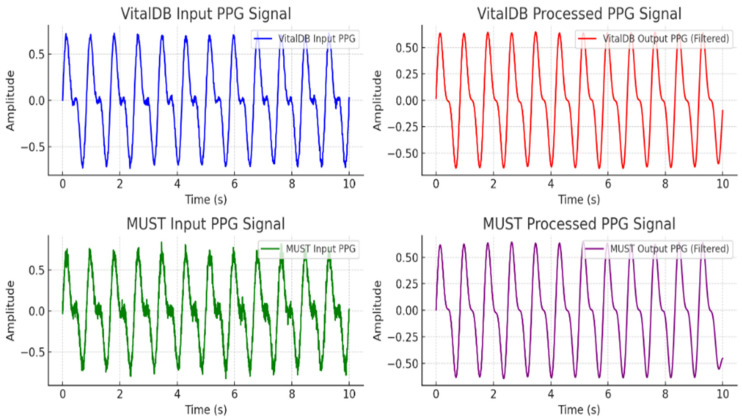
Preprocessing results for both vitalDB and MUST datasets.

**Figure 5:**
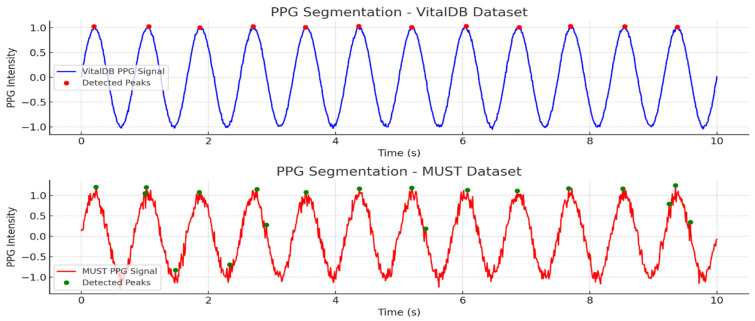
ACBS-based PPG signal segmentation for both vitalDB and MUST datasets.

The system’s assessment metrics portion evaluates the efficacy of the BGM model via numerous essential metrics like MAE (mean absolute error), RMSE (root mean squared error), MSE (mean squared error), MARD (mean absolute relative difference), R^2^ (coefficient of determination) [27], and the Clarke error grid (CEG) [38]. These measures offer quantifiable insights into accuracy, precision, and dependability, facilitating an evaluation of the model’s efficacy in forecasting blood glucose levels. This section evaluates the proposed FL against existing BGM techniques, such as convolutional neural network (CNN) with LSTM-attention, VGG 16, and Resnet 34 [27]. Figure 4 shows the raw and preserved PPG signals from the VitalDB and MUST datasets, highlighting the impact of preprocessing systems. The VitalDB input PPG signal is relatively flawless, displaying distinct peaks that correspond with cardiac cycles, and then the MUST dataset input signal is marked via significant noise and amplitude variations owing to motion irregularities. Next, in preprocessing, the VitalDB processed signal employing CWT-based filtering displays improved peaks and reduced noise, making it more appropriate for feature extraction. Likewise, the processed signal of the MUST dataset, afterward, the use of a CWT, shows a more polished waveform with reduced high-frequency noise. The preprocessing stages are crucial for improving the accuracy of feature extraction and the ensuing prediction of BGLs. The federated learning (FL) configuration table provides a structured overview of the parameters used in this study. Below, Table 2 shows a detailed explanation of each parameter and its significance:

Figure 5 illustrates the segmentation findings utilizing adaptive cycle-based segmentation (ACBS) for the VitalDB and MUST datasets. The VitalDB database displays a largely flawless PPG waveform, so that ACBS accurately identifies 11 cardiac cycles, indicated by red peaks. However, the MUST dataset has a more erratic PPG signal, leading to the detection of 18 cycles, represented by the green peaks. The approach responds to fluctuating heart rates and noise levels via dynamically segmenting the signal at essential peak points, hence assuring precise cycle extraction for subsequent analysis.

Table 3 presents a comprehensive comparison of the extracted features from the VitalDB and MUST datasets, emphasizing the time-domain, frequency-domain, and nonlinear attributes of PPG signals. In the time domain, characteristics like pulse breadth, pulse amplitude, and inter-pulse interval demonstrate fluctuations affected by physiological factors in patient data. Frequency-domain characteristics, such as power spectral density and dominant frequency, highlight variations in cardiovascular activity among the datasets. Nonlinear measures, including fractal dimension and entropy, encapsulate the complexity and variety of PPG data. The MUST dataset has elevated HRV (heart rate variability) and entropy values, indicating increased signal variety, while VitalDB displays more uniform pulse waveform sequences. The collected characteristics are essential inputs for FL models, improving the precision of BGL prediction and categorization.

Table 4 displays the chosen features refined by PSO to improve classification accuracy while decreasing redundancy from the VitalDB and MUST databases. In the VitalDB dataset, 7 of the 10 selected features were utilized, whereas Slope of Downstroke, Inter-Pulse Interval (IPI), and Poincaré Plot SD2 were omitted, indicating their reduced impact on predictive accuracy. In contrast, the MUST dataset retained 9 of the 10 features, omitting Poincaré Plot SD1, which signifies its minimal impact. The PSO-based feature selection significantly improved accuracy.

**Figure 6:**
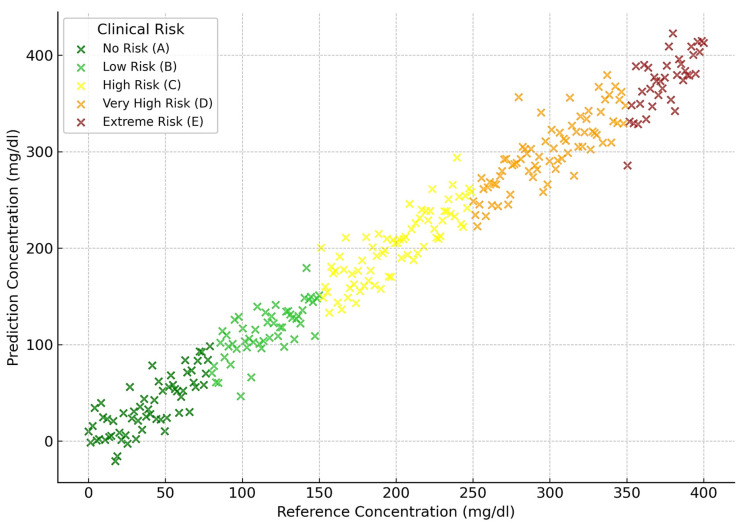
FL predictions-based valuation of clinical risk levels.

**Figure 7:**
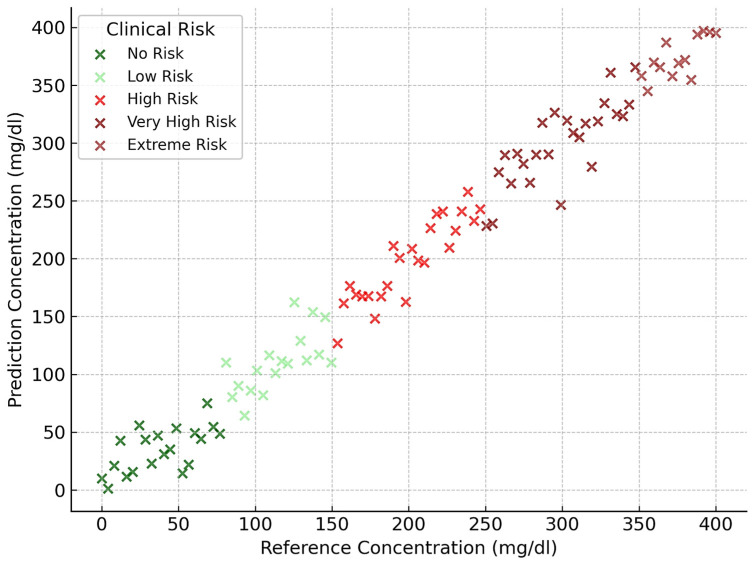
ResNet 34-based valuation of clinical risk levels.

**Figure 8:**
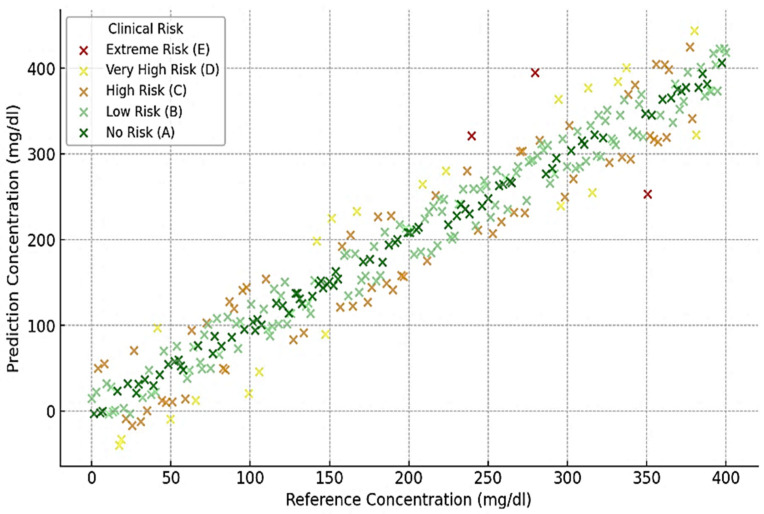
VGG 16-based valuation of clinical risk levels.

**Figure 9:**
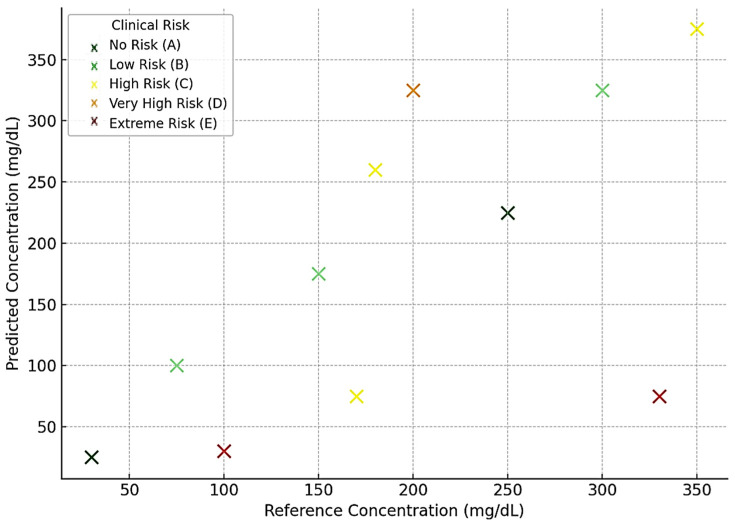
CNN-LSTM-Attention-based valuation of clinical risk levels.

Figure 6, Figure 7, Figure 8 and Figure 9 depict the clinical risk valuation employing the CEG through categorizing zones with data opinions for the assessment sets of dissimilar BGM classification methodologies. These graphs illustrate the correlation between expected and benchmark values, illustrating the spread of forecasts throughout CEG zones to underscore each model’s accuracy and prospective areas for enhancement. Furthermore, they deliver an exhaustive examination of loss measures, providing insights into performance patterns, convergence tendencies, and overall improvements throughout the training process.

**Figure 10:**
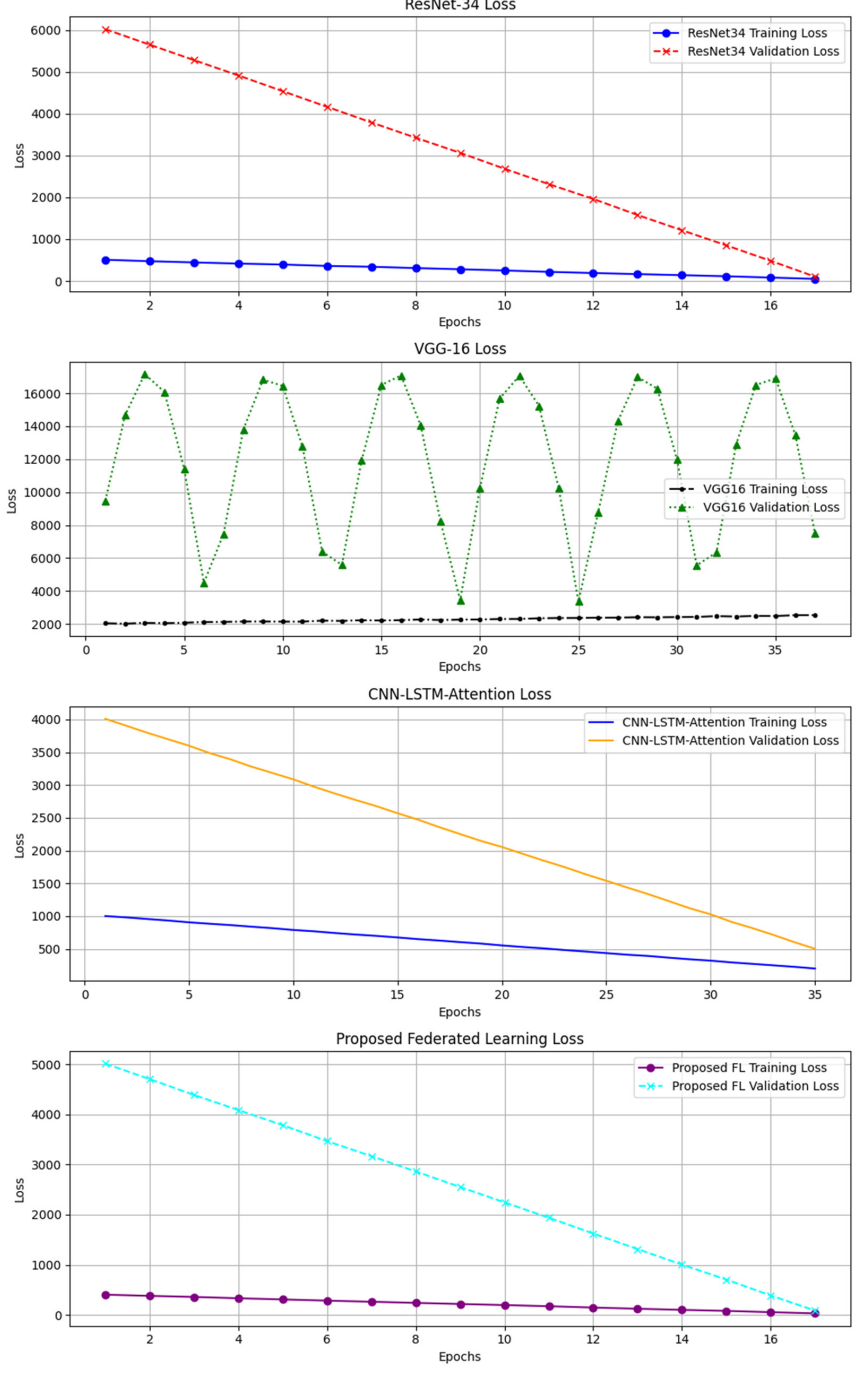
The training and validation loss rates for four BGM schemes.

Figure 10 presents the training and validation loss rates for four models, such as the proposed FL, ResNet34, VGG16, and CNN-LSTM-attention. Each graph depicts the decline of loss over epochs, indicating the progression of model learning. The training loss constantly declines; nevertheless, the validation loss remains high due to overfitting, data variability, or challenges in generalization. The proposed FL demonstrates a steady decline, indicating its effectiveness in decentralized training. The image depicts the performance trends and discrepancies among various deep-learning models.

**Figure 11:**
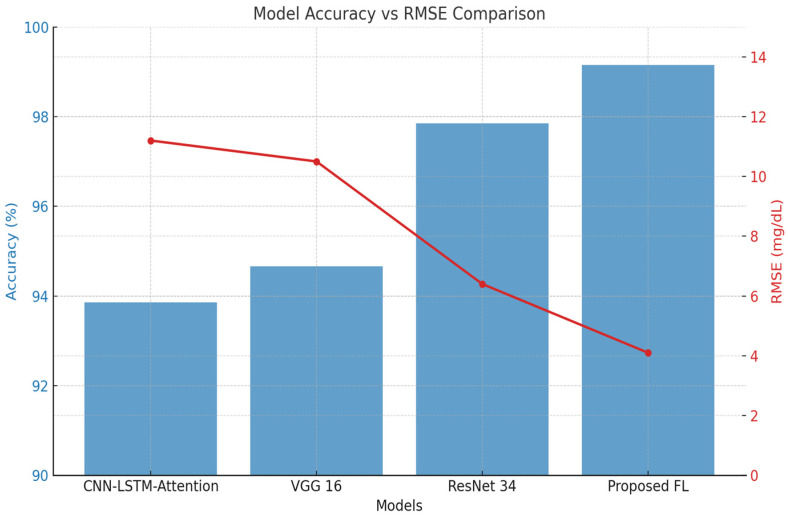
Accuracy and RMSE comparison of proposed FL with baseline models.

Using accuracy and RMSE as the primary assessment measures, Figure 11 compares the performance of four models: CNN-LSTM-attention, VGG 16, ResNet 34, and the proposed federated learning (FL) framework. With an accuracy of 99.15%, the proposed FL model outperforms CNN-LSTM-attention (93.86%), VGG 16 (94.66%), and ResNet 34 (97.85%). In comparison to CNN-LSTM-attention (11.2 mg/dL), VGG 16 (10.5 mg/dL), and ResNet 34 (6.4 mg/dL), it simultaneously logs the lowest RMSE of 4.1 mg/dL, suggesting the least prediction error. The FL model’s capacity to learn from decentralized data sources while maintaining data privacy, in conjunction with efficient feature selection by PSO, is responsible for this better performance. The overall improvements in accuracy and error reduction highlight the model’s resilience and usefulness in situations involving non-invasive BGM.

Table 5 presents a detailed evaluation of BGM models utilizing classification techniques on the VitalDB test database. It presents essential evaluation measures, including MAE, RMSE, MARD, MSE, and R^2^, facilitating a precise valuation for every model’s precision and error performance. It provides significant insights into the expected precision and error estimation of the algorithms by analyzing these parameters across various segmentation methods. The proposed federated learning (FL) model surpasses conventional deep learning methods (ResNet 34, VGG 16, and CNN-LSTM-attention) in PPG-based BGM by attaining superior accuracy, enhanced clinical dependability, and increased predictive consistency. It achieves the lowest RMSE (25.6 mg/dL), MAE (17.6 mg/dL), and MSE (734.5 mg/dL^2^), hence guaranteeing minimum prediction errors. Moreover, its minimal MARD (13.4%) improves clinical acceptability by diminishing erroneous warnings for hypoglycemia or hyperglycemia. The R^2^ value of 0.38 indicates enhanced prediction reliability relative to other models. The FL model is a practical selection for non-invasive BGM, improving accuracy while preserving data privacy in practical digital health applications.

The CEGA is a consistent method for evaluating the precision of BGM by pitting their measurements with the values of reference. The grid is partitioned into five regions according to the degree of variation. Region A has values of exceptional accuracy, facilitating precise glucose monitoring and informed decisions regarding treatment. Region B includes values that demonstrate significant variability without causing adverse treatment effects. In contrast, Regions C, D, and E demonstrate considerable errors, making reliance on them potentially lead to superfluous or hazardous treatments. To be deemed credible, a BGM must have the bulk of its measurements inside Regions A or B of the CEGA.

Table 6 delineates the distribution of forecasts across CEG zones for four blood glucose monitoring (BGM) models: The proposed federated learning (FL) model, ResNet-34, VGG-16, and CNN-LSTM-attention for the VitalDB dataset. The proposed FL model surpasses its counterparts in accuracy, achieving 72.8% in Zone A, just exceeding ResNet-34 (71.6%) and markedly outperforming VGG-16 (52.3%) and CNN-LSTM-attention (56.6%). Furthermore, it exhibits the lowest proportion of predictions in Zone B (17.6%), in contrast to ResNet-34 (26.5%), VGG-16 (45.5%), and CNN-LSTM-attention (40.1%), signifying fewer minor discrepancies. In Zone C, all models exhibit negligible errors, with the proposed FL model at 0.05%, marginally exceeding ResNet-34 (0.03%), VGG-16 (0.02%), and CNN-LSTM-attention (0%). Zone D, indicative of substantial errors, is smaller for the FL model (2.0%) in comparison to ResNet-34 (2.4%), VGG-16 (2.5%), and CNN-LSTM-attention (2.8%). Moreover, in Zone E (critical mistakes), the FL model has the lowest error rate (0.006%), rendering it the most secure among all models. The results underscore the higher accuracy, reduced error rates, and increased reliability of the FL technique and precise BGM while maintaining data privacy and adaptability across various healthcare environments.

This system also assesses the proposed optimal FL model intended for ACBS segmentation utilizing the MUST database. These data consist of observations from 23 persons, each contributing multiple signal segments along with their corresponding BGL test findings. To ensure consistency, these signals were first resampled to a frequency of 100 Hz. The chosen segments encompass systolic and diastolic peak periods together, as they signify essential physiological processes pertinent to this investigation. Table 7 shows the evaluation metrics of the FL method, whereas Table 8 illustrates the outcomes of the CEG analysis.

Table 7 assesses the performance of four models: Proposed FL, ResNet-34, VGG-16, and CNN-LSTM-attention on the MUST dataset. The proposed FL model surpasses the others, attaining the lowest MAE (14.1 mg/dL), RMSE (19.1 mg/dL), MSE (388 mg/dL^2^), and MARD (12.3%), indicating superior accuracy and little departure from reference values. Conversely, ResNet-34, VGG-16, and CNN-LSTM-attention exhibit elevated error rates, with CNN-LSTM-Attention demonstrating the greatest RMSE (25.4 mg/dL) and MSE (588.3 mg/dL^2^), signifying higher prediction discrepancies. The enhanced efficacy of the proposed FL model is due to its decentralized learning framework, which improves generalization by leveraging varied data sources while maintaining privacy protection. Furthermore, its refined feature selection and adaptive learning algorithms enhance accuracy and minimize errors, rendering it a highly dependable and effective solution for BGM with the MUST dataset.

Table 8 delineates the distribution of CEG zones for four models like, proposed FL, ResNet-34, VGG-16, and CNN-LSTM-attention, assessing their precision in BGM. The proposed FL model exhibits the best accuracy, achieving 76.8% of predictions in Zone A, signifying extremely trustworthy readings, surpassing ResNet-34 (72.4%), VGG-16 (69.3%), and CNN-LSTM-attention (60.6%). The proposed FL model has 22.7% of its predictions in Zone B, indicating minor deviations that do not substantially affect clinical decisions, akin to ResNet-34 (24.3%). In contrast, VGG-16 (45.5%) and CNN-LSTM-attention (40.1%) demonstrate a higher percentage in this zone, implying better variability. The proposed FL model exhibits no predictions in Zones C, D, and E, signifying an absence of significant errors, though alternative models demonstrate misclassifications—VGG-16 and CNN-LSTM-Attention present elevated error rates, with CNN-LSTM-attention recording 6.3% in Zone C and 0.7% in Zone E, indicating possible clinical risks. The results underscore the enhanced performance, reduced error rates, and increased dependability of the proposed FL model, rendering it a more efficacious alternative for BGM.

**Figure 12:**
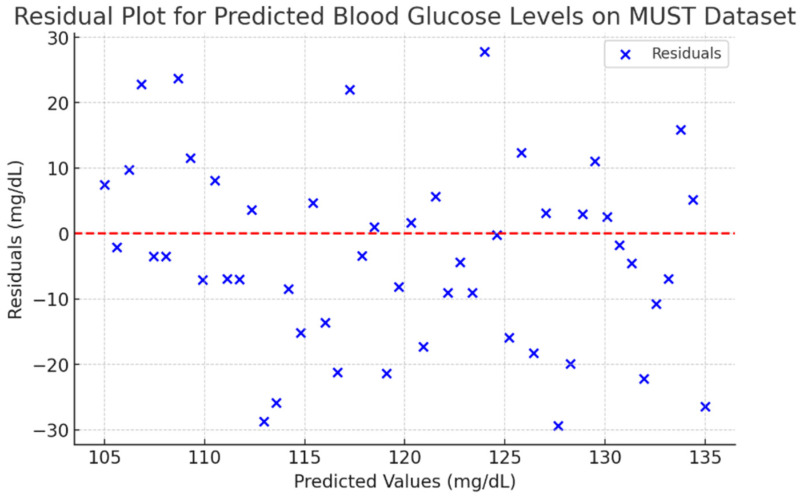
Predicted BGL on MUST dataset.

**Figure 13:**
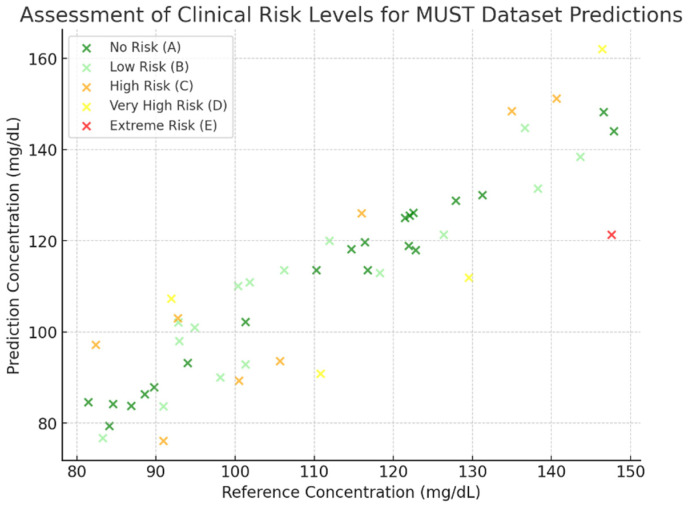
Valuation of clinical risk for MUST data.

The residual plot for predicted BGLs with the MUST dataset demonstrates the discrepancies between expected and real values displayed contrary to the anticipated outcomes. This visualization facilitates the assessment of the model’s correctness, identification of potential biases, and the consistency of the model’s predictions, which is evaluated and illustrated in Figure 12, while the clinical risk assessment for the MUST data is presented in Figure 13.

Table 9 and Table 10 present a comparative analysis of the performance of four models such as the proposed FL model, ResNet-34, VGG-16, and CNN-LSTM-attention, assessed based on accuracy, recall, precision, sensitivity, specificity, and F1-score metrics for both the vitalDB and MUST datasets. The proposed FL model regularly surpasses the others, attaining the highest accuracy (99.15 and 99.31%), sensitivity (98.41 and 98.65%), specificity (98.37 and 99.14%), recall (98.57 and 98.75%), precision (99.25 and 99.45%), and F1-score (98.34 and 99.12%) for both vitalDB and MUST datasets. These results highlight its exceptional classification effectiveness, minimizing both false positives and negatives. The superior performance of the proposed FL model is due to its centralized learning approach, which facilitates the use of diverse datasets across multiple devices without the need to share raw data centralization, thus enhancing generalization and robustness. Furthermore, FL increases flexibility by integrating real-world variations in patient data, leading to more accurate and personalized forecasts. The combination of improved feature selection and advanced DL models enhances accuracy, providing it with a highly precise and effective choice for ACBS segmentation and BGM.

## 5. Conclusions

This study demonstrates the transformative potential of federated learning (FL) in PPG-based blood glucose monitoring (BGM), offering a scalable, secure, and clinically reliable method for non-invasive diabetes treatment. Traditional centralized machine-learning approaches have major difficulties because of data privacy challenges, regulatory limitations, and the necessity for extensive, diverse datasets. The proposed FL examines these difficulties by facilitating joint training of a global model across several healthcare institutions without the transfer of raw patient data, thereby ensuring data confidentiality and compliance with regulatory norms. The suggested method utilizes continuous wavelet transform (CWT) for signal preprocessing to remove baseline drift and noise, and applies particle swarm optimization (PSO) for optimal feature selection, thereby enhancing the precision of classification. The proposed FL-based BGM system has demonstrated superior performance related to conventional deep learning models, including ResNet-34, VGG-16, and CNN-LSTM-attention. The model’s efficacy was evaluated using multiple metrics, including MAE, RMSE, MSE, MARD, R^2^, and Clarke error grid analysis (CEGA) on the VitalDB and MUST datasets. The FL model achieved the lowest error rates and highest accuracy, ensuring reliable BGL predictions. It attained RMSE values of 25.6 mg/dL (VitalDB) and 19.1 mg/dL (MUST), with improved MARD of 13.4% and 12.3%, respectively. Moreover, it had the highest prediction rates in Zone A of the CEGA, with 72.8% for VitalDB and 76.8% for MUST, indicating improved clinical dependability. Real-time PPG data collection and continuous GL prediction are made possible by the combination of the FL model into wearable technology, such as smartwatches or sensor-embedded patches, for everyday practical application. To ensure privacy and lower latency, these wearables can use edge computing to interpret signals locally before sending encrypted model changes to the federated network. Creating standardized datasets for a range of the population, encouraging institutional partnerships for safe data exchange, and establishing federated analytics should be the main goals of future non-invasive glucose monitoring research. Prediction accuracy can be further increased by investigating new sensors, multi-modal signal fusion (such as PPG with ECG), and adaptive algorithms. The clinical translation and inclusive acceptance of these technologies will be facilitated by an emphasis on ethical AI practices and regulatory compliance.

## Figures and Tables

**Table 1:** Research gap between non-invasive blood glucose monitoring schemes.

Authors	Method Name	Advantages	Disadvantages
Nie et al., [19]	PCR, PLS, SVR, RFR	This method allows painless and convenient BGM without the need for finger pricks.	The precision may be impacted by environmental factors such as illumination conditions and dermal changes.
Zeynali et al., [27]	VGG16, ResNet34, and a hybrid CNN-LSTM	The new 1-s PPG signal method makes blood glucose measurement more accurate and faster, providing results in just 6.4 s.	The model’s accuracy may be affected by movements and changes in body conditions, making it less reliable in real-life use.
Chen, S. et al. [28]	MvCFT	It is a painless and easy way to check blood sugar without pricking the skin.	The results may not always be accurate due to body movements or other factors.
Rostami, A., et al., [29]	LSTM	Enables real-time stress detection on low-power wearable devices.	Accuracy may be affected by motion artifacts and individual variations.
Satter et al., [30]	CatBoost, XGBoost, LightGBM, and random forest	The proposed system makes BGM easier and painless, avoiding finger pricks and improving patient comfort.	The system’s accuracy can be affected by various influencing factors, such as skin tone, lighting conditions, and motion, which are less dependable.
Chowdhury et al., [31]	MMG-net	Provides a non-invasive, comfortable, and accurate way to BGM using wearable devices, reducing the need for finger pricks.	The system’s reliability may be contingent upon external circumstances like sensor quality, user adherence, and variations in individual physiology.
Susana et al., [32]	SVM	SVM provides high accuracy (91.3%) for BGM, making it a reliable classifier.	SVM has a longer training time and may require significant computational resources for large datasets.
Vargová et al., [33]	SVM and RF	SVM and RF provide reliable accuracy for classifying BGLs, making them useful for non-invasive monitoring.	Both models require careful feature selection and tuning, which can be time-consuming and computationally intensive.
Susana et al., [34]	ML	ML improves BGM accuracy by efficiently analyzing PPG signals for diabetes detection.	Optimal performance necessitates high-quality data and appropriate feature selection, which can be time-intensive.
Yen et al., [35]	BPNN	BPNN discerns intricate patterns inside data, enhancing the precision of accuracy.	BPNN frequently necessitates multiple rounds to reduce error, resulting in extended training durations.

**Table 2:** Federated Learning Configuration Parameters.

Parameters	Value	Description
Number of clients	5	Simulated healthcare institutions participating in FL
Local model architecture	3-layer LSTM	Deep learning model for blood glucose prediction
LSTM Units	64 per layer	Number of units in each LSTM layer
Activation function	ReLU	Used in the hidden layer
Dropout rate	0.3	To prevent overfitting
Optimizer	Adam	Optimization algorithm for training
Learning rate	0.001	Controls the step size during training
Batch size	32	Number of samples per training batch
Aggregation method	Federated averaging (FedAvg)	Method to combine local model updates
Number of rounds	20	Global communication rounds in FL
Privacy consideration	No raw data sharing	Only model weights are exchanged

**Table 3:** Extracted feature from the PPG signal for both VitalDB and MUST databases.

Categorization of Feature	Extracted Features	VitalDB Feature Extraction Values	MUST Feature Extraction Values
Time domain	Pulse width (s)	0.85 ± 0.07	0.91 ± 0.08
	Pulse amplitude (a.u.)	1.25 ± 0.10	1.42 ± 0.12
	Inter-pulse interval (IPI) (s)	0.92 ± 0.05	0.88 ± 0.04
	Area under the curve (AUC)	2.65 ± 0.18	3.02 ± 0.21
	Slope of upstroke (a.u./s)	0.93 ± 0.05	0.89 ± 0.04
	Slope of downstroke (a.u./s)	0.74 ± 0.06	0.83 ± 0.07
Frequency domain	Heart rate variability (HRV) (ms)	52.4 ± 3.51	48.9 ± 3.21
	Power spectral density (PSD) (dB/Hz)	15.8 ± 1.22	17.3 ± 1.41
	Dominant frequency (Hz)	1.13 ± 0.09	1.25 ± 0.10
Nonlinear feature	Fractal dimension (FD)	1.42 ± 0.06	1.51 ± 0.07
	Entropy	0.85 ± 0.03	0.93 ± 0.04
	Poincaré plot SD1 (ms)	18.5 ± 1.41	20.1 ± 1.62
	Poincaré plot SD2 (ms)	47.3 ± 2.92	50.7 ± 3.12

**Table 4:** PSO-based selected features from PPG signal for both VitalDB and MUST databases.

Categorization of Feature	Extracted Features	VitalDB Dataset	MUST Dataset
Time domain	Pulse width (s)	Selected	Selected
	Pulse amplitude (a.u.)	Selected	Selected
	Inter-pulse interval (IPI) (s)	Not selected	Selected
	Area under the curve (AUC)	selected	Selected
	Slope of upstroke (a.u./s)	Selected	Selected
	Slope of downstroke (a.u./s)	Not selected	selected
Frequency domain	Heart rate variability (HRV) (ms)	Not selected	selected
	Power spectral density (PSD) (dB/Hz)	Selected	Selected
	Dominant frequency (Hz)	Selected	Selected
Nonlinear feature	Fractal dimension (FD)	Selected	Selected
	Entropy	Selected	Selected
	Poincaré plot SD1 (ms)	Selected	Not Selected
	Poincaré plot SD2 (ms)	Not Selected	Selected

**Table 5:** Performance evaluation among BGM schemes for VitalDB dataset.

Metrics	Proposed FL	ResNet 34	VGG 16	CNN-LSTM-Attention
RMSE (mg/dL)	25.6	27.4	35.7	42.1
MAE (mg/dL)	17.6	18.3	26.6	27.5
$\mathrm{MSE}\;{\left(\frac{mg}{dL}\right)}^{2}$	734.5	755.5	1223.2	1738.2
MARD (%)	13.4	15.45	22.8	20.51
R^2^	0.38	0.40	−0.7	0.00

**Table 6:** Clarke error grid analysis among BGM schemes in the VitalDB dataset.

Zones (%)	Proposed FL	ResNet 34	VGG 16	CNN-LSTM-Attention
A	72.8	71.6	52.3	56.6
B	17.6	26.5	45.5	40.1
C	0.05	0.03	0.02	0
D	2.0	2.4	2.5	2.8
E	0.006	0.003	0	0

**Table 7:** Performance evaluation among BGM schemes for the MUST dataset.

Metrics	Proposed FL	ResNet 34	VGG 16	CNN-LSTM-Attention
RMSE (mg/dL)	19.1	20.1	21.1	26.6
MAE (mg/dL)	14.1	14.8	17	17.5
MSE ${\left(\frac{mg}{dL}\right)}^{2}$	388	394.2	410.5	588.3
MARD (%)	12.3	13.1	15.4	13.4

**Table 8:** Clarke error grid analysis among BGM schemes MUST dataset.

Zones (%)	Proposed FL	ResNet 34	VGG 16	CNN-LSTM-Attention
A	76.8	72.4	69.3	60.6
B	22.7	24.3	45.5	40.1
C	0	3.6	5.1	6.3
D	0	0.1	0.5	1
E	0	0	0.3	0.7

**Table 9:** Overall prediction performance results among BGM schemes for the VitalDB dataset.

Methods	Accuracy	Sensitivity	Specificity	Recall	Precision	F1 Score
CNN-LSTM-attention	93.86	89.71	94.04	92.12	88.92	91.03
VGG 16	94.66	91.35	91.34	94.23	93.62	93.73
ResNet 34	97.85	97.82	96.55	96.57	97.21	96.23
Proposed FL	99.15	98.41	98.37	98.57	99.25	98.34

**Table 10:** Overall prediction performance results among BGM schemes for the MUST dataset.

Methods	Accuracy	Sensitivity	Specificity	Recall	Precision	F1 Score
CNN-LSTM-attention	94.15	90.10	94.51	92.88	89.16	92.03
VGG 16	95.66	91.92	96.05	95.65	94.83	95.73
ResNet 34	98.12	97.14	98.03	96.89	97.66	97.23
Proposed FL	99.31	98.65	99.14	98.75	99.45	99.12

## Data Availability

Data are contained within the article.

## Abbreviations

The following abbreviations are used in this manuscript:

BGLs	blood glucose levels
PPG	photoplethysmography
DNNs	deep neural networks
ML	machine learning
FL	federated learning
CWT	continuous wavelet transform
ACBS	adaptive cycle-based segmentation
PSO	particle swarm optimization
RMSE	root mean square error
CEGA	Clarke error grid analysis
MvCFT	multi-view cross-fusion transformer
MSWF	multi-size weighted fitting
CVFF	cross-view feature fusion
LSTM	long short-term memory
PTQ	post-training quantization
QAT	quantization aware training
IMF	intrinsic mode functions
EMD	empirical mode decomposition
MAE	mean absolute error
MSE	mean square error
EDA	electrodermal activity
PLS	partial least squares
PCR	principal component regression
RFR	random forest regression
SVR	support vector regression
SVM	support vector machine
RF	random forest
BPNN	back-propagation neural network
PCA	principal component analysis
ICWT	inverse continuous wavelet transform
FA	federated averaging
MTL	multi-task learning
CCE	categorical cross-entropy
MARD	mean absolute relative difference
CNN	convolutional neural network
HRV	heart rate variability
IPI	inter-pulse interval
AUC	area under the curve
PSD	power spectral density
FD	fractal dimension
